# Towards harmonized laboratory methodologies in veterinary clinical bacteriology: outcomes of a European survey

**DOI:** 10.3389/fmicb.2024.1443755

**Published:** 2024-10-10

**Authors:** Tom Koritnik, Iskra Cvetkovikj, Flavia Zendri, Shlomo Eduardo Blum, Serafeim Christos Chaintoutis, Peter A. Kopp, Cassia Hare, Zrinka Štritof, Sonja Kittl, José Gonçalves, Irena Zdovc, Erik Paulshus, Andrea Laconi, David Singleton, Fergus Allerton, Els M. Broens, Peter Damborg, Dorina Timofte

**Affiliations:** ^1^Department for Public Health Microbiology Ljubljana, Centre for Medical Microbiology, National Laboratory of Health, Environment and Food, Ljubljana, Slovenia; ^2^Department of Microbiology and Immunology, Faculty of Veterinary medicine-Skopje, Ss Cyril and Methodius University in Skopje, Skopje, Republic of North Macedonia; ^3^Department of Veterinary Anatomy, Physiology and Pathology, Institute of Infection, Veterinary and Ecological Sciences, School of Veterinary Science, Leahurst Campus, University of Liverpool, Neston, United Kingdom; ^4^ESCMID Study Group for Veterinary Microbiology (ESGVM), Basel, Switzerland; ^5^Department of Bacteriology and Mycology, Kimron Veterinary Institute, Bet Dagan, Israel; ^6^Diagnostic Laboratory, School of Veterinary Medicine, Faculty of Health Sciences, Aristotle University of Thessaloniki, Thessaloniki, Greece; ^7^IDEXX Vet Med Labor GmbH, Kornwestheim, Germany; ^8^Department of Veterinary Medicine, University of Cambridge, Cambridge, United Kingdom; ^9^Department of Microbiology and Infectious Diseases with Clinic, Faculty of Veterinary Medicine, University of Zagreb, Zagreb, Croatia; ^10^Department of Infectious Diseases and Pathobiology, Institute of Veterinary Bacteriology, University of Bern, Bern, Switzerland; ^11^MARE−Marine and Environmental Sciences Centre, ARNET−Aquatic Research Network Associate Laboratory, NOVA School of Science and Technology, NOVA University Lisbon, Caparica, Portugal; ^12^Veterinary Faculty of Ljubljana, Institute of Microbiology and Parasitology, Ljubljana, Slovenia; ^13^Department of Analysis and Diagnostics, Microbiology, Norwegian Veterinary Institute, Ås, Norway; ^14^Department of Comparative Biomedicine and Food Science, University of Padua, Legnaro, Italy; ^15^Willows Veterinary Centre and Referral Service, Shirley, United Kingdom; ^16^Department of Biomolecular Health Sciences, Faculty of Veterinary Medicine, Utrecht University, Utrecht, Netherlands; ^17^Department of Veterinary and Animal Sciences, Faculty of Health and Medical Sciences, University of Copenhagen, Frederiksberg, Denmark

**Keywords:** veterinary clinical bacteriology, bacterial culture, bacterial identification, antimicrobial susceptibility testing, harmonization, methodologies, ENOVAT

## Abstract

**Introduction:**

Veterinary clinical microbiology laboratories play a key role in antimicrobial stewardship, surveillance of antimicrobial resistance and prevention of healthcare associated-infections. However, there is a shortage of international harmonized guidelines covering all steps of veterinary bacterial culture from sample receipt to reporting.

**Methods:**

In order to gain insights, the European Network for Optimization of Veterinary Antimicrobial Treatment (ENOVAT) designed an online survey focused on the practices and interpretive criteria used for bacterial culture and identification (C&ID), and antimicrobial susceptibility testing (AST) of animal bacterial pathogens.

**Results:**

A total of 241 microbiology laboratories in 34 European countries completed the survey, representing a mixture of academic (37.6%), governmental (27.4%), and private (26.5%) laboratories. The C&ID turnaround varied from 1 to 2 days (77.8%) to 3–5 days (20%), and 6– 8 days (1.6%), with similar timeframes for AST. Individual biochemical tests and analytical profile index (API) biochemical test kits or similar were the most frequent tools used for bacterial identification (77% and 56.2%, respectively), followed by PCR (46.6%) and MALDI-TOF MS (43.3%). For AST, Kirby-Bauer disk diffusion (DD) and minimum inhibitory concentration (MIC) determination were conducted by 43.8% and 32.6% of laboratories, respectively, with a combination of EUCAST and CLSI clinical breakpoints (CBPs) preferred for interpretation of the DD (41.2%) and MIC (47.6%) results. In the absence of specific CBPs, laboratories used human CBPs (53.3%) or veterinary CBPs representing another body site, organism or animal species (51.5%). Importantly, most laboratories (47.9%) only report the qualitative interpretation of the result (S, R, and I). As regards testing for AMR mechanisms, 48.5% and 46.7% of laboratories routinely screened isolates for methicillin resistance and ESBL production, respectively. Notably, selective reporting of AST results (i.e. excluding highest priority critically important antimicrobials from AST reports) was adopted by 39.5% of laboratories despite a similar proportion not taking any approach (37.6%) to guide clinicians towards narrower-spectrum or first-line antibiotics.

**Discussion:**

In conclusion, we identified a broad variety of methodologies and interpretative criteria used for C&ID and AST in European veterinary microbiological diagnostic laboratories. The observed gaps in veterinary microbiology practices emphasize a need to improve and harmonize professional training, innovation, bacterial culture methods and interpretation, AMR surveillance and reporting strategies.

## Introduction

Microbiological diagnostic laboratories play an important role in antimicrobial stewardship, since results may impact the decision to treat with an antimicrobial agent and the drug selected. Optimization and standardization of the diagnostic process, including all steps from sample collection to reporting of the results, are key factors to obtain reproducible and reliable results that can support evidence-based therapeutic decisions by clinicians.

In human clinical microbiology, several international manuals (UK Standards for Microbiology Investigations [UK SMIs], 2023) and standards have existed for many years. For instance, the ISO 15189 standard developed by the International Organization for Standardization that specifies requirements for quality and competence in medical laboratories is recognized and implemented throughout the world (ISO 15189:2022(en), 2022). The ISO/IEC 17025:2017 standard (ISO/IEC 17025:2017(en), 2017) setting the general requirements for the competence of testing and calibration laboratories and Good Laboratory Practice (GLP) policies may be implemented by veterinary microbiology laboratories for demonstration of competency to carry out high quality and accurate laboratory testing. However, with a few notable exceptions such as the WOAH Manual of Diagnostic Tests and Vaccines for Terrestrial Animals (WOAH, 2023) and standards for AST of veterinary pathogens (CLSI VET02, 2021; CLSI VET01, 2024; CLSI VET01S, 2024; CLSI VET09, 2024), similar standards and guidelines have not been developed specifically for veterinary diagnostic laboratories. Although standards developed for human microbiology can be routinely used by veterinary diagnostic laboratories (Cornaglia et al., 2012; Leber and Burnham, 2023), they are not directly transferable and applicable to veterinary laboratories, for instance due to the existence of animal pathogens with specific growth requirements (Guardabassi et al., 2017).

Adding to this problem, standardized training of veterinary clinical microbiologists is still in its infancy. The establishment of the EBVS recognized European College for Veterinary Microbiology (ECVM)^1^ in 2016 was a major step forward that should lead to more well-trained veterinary microbiologists. Also, VetCAST (Toutain et al., 2017; EUCAST, 2015) (subcommittee of EUCAST) and CLSI-VAST (Feßler et al., 2023; Veterinary Antimicrobial Susceptibility Testing [VAST], 2024) (subcommittee of CLSI) are producing veterinary-specific manuals and clinical breakpoints. However, clinical breakpoints for many antimicrobial agent - animal species - body site - pathogen combinations are still lacking and other steps of the diagnostic process beyond AST, such as bacterial culture and identification methods, selection of relevant isolates for AST and reporting approaches, remain poorly addressed for veterinary diagnostic laboratories.

Taken together, these shortcomings in veterinary diagnostic microbiology, combined with any technical errors that may conceivably occur in routine bacteriological diagnostics (Rampacci et al., 2021; Cuénod et al., 2023), impose a risk of poor-quality results and potential large differences between laboratories, which may lead to suboptimal treatment and subsequent selection of antimicrobial resistance in animal patients. Furthermore, the lack of surveillance harmonization poses considerable challenges to accurately quantify AMR, understanding its dynamics and implementing effective strategies to mitigate its impact.

As part of the Cooperation in Science and Technology (COST) actions, the European Network for Optimization of Veterinary Antimicrobial Treatment (ENOVAT−CA18217)^2^ has been established. A key aim of ENOVAT was to refine and harmonize veterinary microbiological diagnostic procedures. This survey-based ENOVAT study, was intended to gain insight into the current practices used by European diagnostic laboratories, with a focus on the methodologies and interpretative criteria used for bacterial culture and AST of animal clinical specimens. Information about such practices provides the foundation for identifying knowledge gaps and areas to prioritize during future works and to harmonize veterinary microbiology procedures.

## Materials and methods

### Survey design and distribution

An ethical approval for undertaking an online survey was secured through the University of Liverpool Research Ethics and Integrity Office (VREC958). The survey was designed by a panel of ENOVAT members including veterinary clinicians, microbiologists and epidemiologists representing seven different countries. The survey was designed and distributed via the online tool SurveyMonkey (SurveyMonkey Inc., USA). The survey included 37 questions (Supplementary material), divided into four topics: laboratory information (Section A), methodology related to bacterial culture, identification and susceptibility testing (Section B), results interpretation and reporting (Section C), and surveillance, laboratory data management and further developments (Section D).

Prior to its launch, the survey was piloted among ENOVAT members to pinpoint essential concerns, detect any potentially confusing questions and incorporate additional suggestions obtained during this pilot phase.

The survey was distributed in February 2021 via the ENOVAT Network as a weblink advertised on the ENOVAT webpage, social media and sent via the consortium channels. Country representatives from the ENOVAT Network have been actively involved in dissemination of the survey in their respective countries through engagement with veterinary national bodies, agencies and partners. Participation to the survey was on a voluntary basis, and responses were collected until mid-August 2021.

**FIGURE 1:**
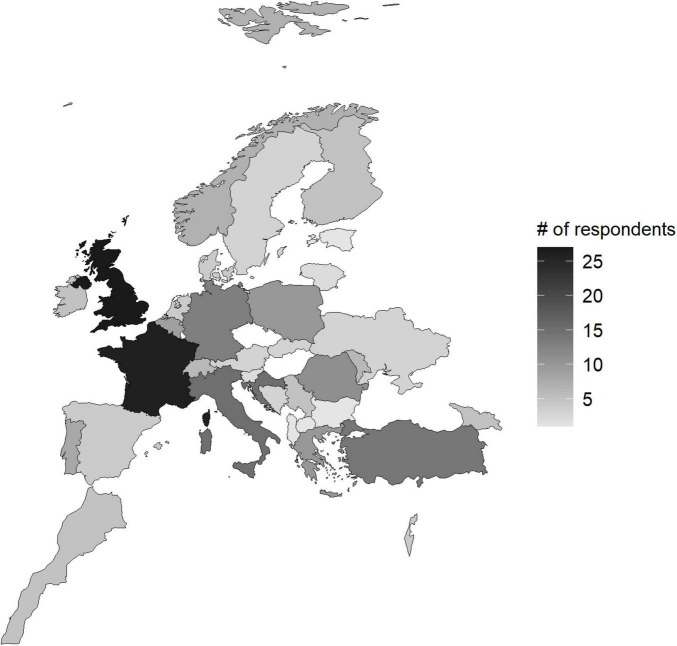
Geographic distribution of survey responders across Europe.

The presented data have not been adjusted according to parameters like the accurate number of laboratories per participating country, scale of animal production, number of companion animals and other factors. Rather, we aimed to present the raw data gathered from the participating laboratories, as described below. As the number of participating laboratories (Supplementary Table 1 and Figure 1) greatly varied between countries but information on the number of veterinary laboratories in each country is inconsistent, country-specific comparisons and conclusions could not be investigated.

### Methodology for inclusion and analysis of complete and partial responses in the survey

Answers for each question were analysed and responses from laboratories providing full and partial answers were used if appropriate. Partial answers were included under the assumption that some participants were either reluctant or unwilling to disclose data, or uncertain of the answer. Working under this assumption, each question was analysed separately. Partial answers were included in the analysis, except when an answer was not in agreement with a previous response. To exemplify the latter scenario, one participant reported failure to participate in external quality assurance programs and despite this selected one of the optional assurance schemes.

### Statistical analysis

Survey results were exported with the SurveyMonkey export tool and imported to IBM SPSS Statistics 28.0.1.0 (IBM, USA). Descriptive statistics and figures were generated for each survey response if appropriate. Microsoft Excel (Microsoft, USA) was used for certain data sorting tasks if needed. R programming language was used with the RStudio (Allaire, 2012) software for visualization of certain graphs and figures.

## Results

Representatives from 290 laboratories commenced the survey. Nineteen laboratories were automatically excluded, as they declined to consent to participate in the survey (*n* = 4) or indicated that they did not offer bacterial culture and AST (*n* = 15). An additional 30 participants were excluded as they did not answer any further questions despite completing the inclusion criteria. Overall, 241 respondents completed the survey, at least partially; this variability in the number of responses was acceptable as not all questions relating to methodologies or available facilities were applicable to all respondents.

Respondents were from laboratories in a variety of European and two neighbor countries (i.e. Morocco and Israel), most frequently the United Kingdom (27/241, 11.2%), France (26/241, 10.7%), Croatia (15/241, 6.2%), Italy (15/241 6.2%), Germany (13/241, 5.4%) and Romania (11/241, 4.6%) (Figure 1). The complete list of participants by country is available in Supplementary Table 1.

### Section A: laboratory information

**FIGURE 2:**
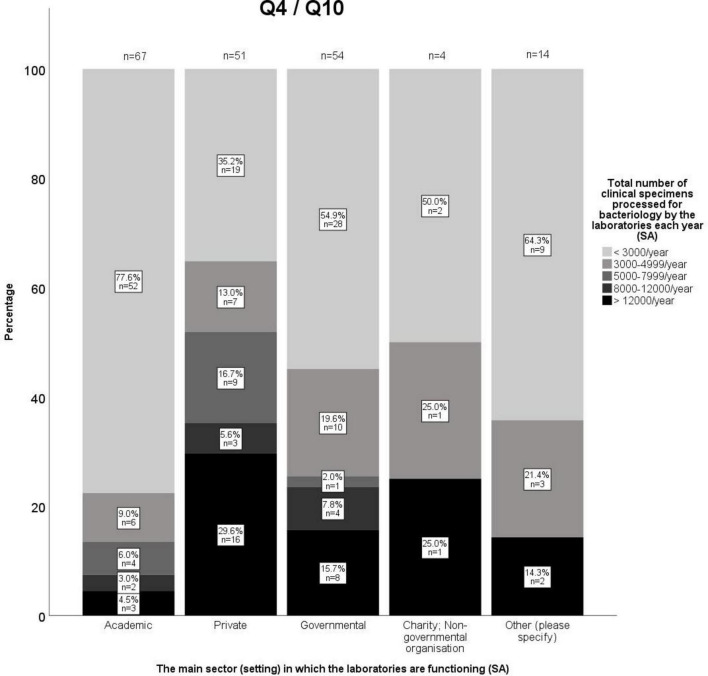
Number of clinical specimens processed for bacteriology by the laboratories based on the main sector in which they are functioning.

An overview of responses to questions concerning laboratory settings and processes is provided in Supplementary Table 2, and a summary is provided in the following text. Most laboratories were situated in academic settings (37.6%), followed by governmental (27.4%) and private (26.5%) settings (Supplementary Table 2). Within the private sector, most laboratories were commercial (65.6%) followed by in-house veterinary practice/hospital laboratories (26.2%). The majority of participating laboratories (90.7%) processed clinical samples from animals, whereas 23.1% and 18.5% processed food or feed (i.e. animal derived food for human consumption or animal feed) and environmental samples, respectively. The majority of laboratories in all settings processed fewer than 3,000 samples per year (Figure 2).

In most laboratories, the microbiology diagnostics team consisted of technical staff (84.9%) and veterinary microbiologists (77.3%). More than half of the laboratories were headed by a veterinary microbiologist (55.7%), followed by a veterinarian/clinician (13%) and microbiologist of non-veterinary background (9.2%) (Supplementary Table 2). The free text responses included in the “Other” category (7%) indicated diverse backgrounds for this leader role, such as veterinary parasitologist, pathologist, engineer, chemist, or financial manager.

Most laboratories (75.1%) provide guidance for optimal specimen collection and management via various routes including: telephone (72.3%), e-mail (57.2%), website information (47.2%) and sample submission forms (41.5%) (Supplementary Table 2).

Quality assurance (QA) was by far less common in academic laboratories compared to laboratories in other settings (Supplementary Table 2). As for the type of QA, 70.5% of all laboratories indicated that they conducted internal QA, primarily in the form of Standard Operating Procedures (90.1%), equipment maintenance and calibration (85.6%), and use of quality control strains (85.6%). Participation in external QA was less common (59.6%) and occurred mostly by taking part in national proficiency testing (76.6%), accreditation from a recognized QA system (e.g. ISO) (68.5%), and external audits (67.6%).

Most of the laboratories reported 1–2 days turnaround time for both bacterial culture and identification (77.8%) and AST (62.7%). Longer turnarounds were less commonly reported by laboratories for both C&ID (20% and 1.6% for 3–5 and 6–8 days, respectively) and AST (32.4% for 3–5 and 4.3% for 6–8 days, respectively).

### Section B. Methodology (bacterial culture, identification and susceptibility testing)

**FIGURE 3:**
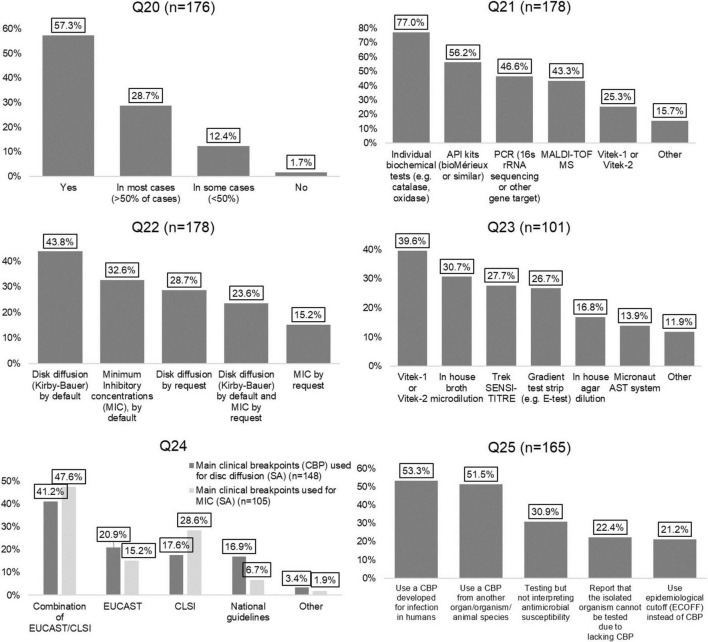
Figures for questions Q20–Q25. Responses regarding the methodology employed by the participating laboratories for bacterial culture and antimicrobial susceptibility testing. Percentages are based on the number of responses to individual questions. MA, multiple answer question; SA, single answer question. Q20–Identification to species level (SA); Q21–Methods for bacterial identification (MA); Q22–AST method provided (MA); Q23–MIC method provided (MA); Q24-1–Main clinical breakpoints (CBP) used for disc diffusion (SA), Q24-2–Main clinical breakpoints used for MIC (SA); Q25–Approach when no species–specific CBPs (MA).

An overview of responses to questions concerning methodology used by laboratories is provided in Figure 3 and Supplementary Table 3. Nearly all laboratories (97.8%) offered aerobic culture, followed by anaerobic (89.3%), microaerophilic (77.0%), and 5–10% CO_2_ enriched culture (71.3%). Only 41.6% of laboratories provided selective culture for one or more of the following target species/phenotypes: *Salmonella* spp., methicillin-resistant *Staphylococcus aureus* (MRSA) and *Staphylococcus pseudintermedius* (MRSP), *Campylobacter* spp., *Listeria* spp., *Yersinia* spp., *Bartonella* spp., *Brucella* spp., *Brachyspira* spp., and *Dermatophilus* spp.

Only three laboratories (1.7%) reported that they do not attempt to identify bacterial isolates at species level, whilst others performed this in all (57.3%), most (28.7%) or some (12.4%) cases. Individual biochemical assays (e.g. catalase, oxidase) for bacterial identification were employed in 77% of laboratories, followed by API kits or similar (56.2%), PCR (46.6%), MALDI-TOF MS (43.3%), and VITEK 1 or the VITEK 2 automated ID/AST instruments (25.3%) (Supplementary Table 4).

For AST, by default most laboratories (43.8%) reported using Kirby-Bauer disk diffusion (DD), followed by minimum inhibitory concentration (MIC) determination (32.6%). In addition, some laboratories only provide DD (28.7%) or MIC (15.2%) results upon request. The most commonly used approach to perform MIC testing was VITEK automated ID/AST instruments (39.6%), followed by in-house broth microdilution (30.7%), Trek Sensititre (27.7%), and gradient test strips (26.7%) (Supplementary Table 4).

More laboratories reported using a combination of EUCAST and CLSI clinical breakpoints (CBPs) for interpreting AST results than using solely EUCAST or CLSI breakpoints. This trend was observed for both DD (41.2%) and MIC testing (47.6%). In the absence of animal species - body site−pathogen specific CBPs for one or more antimicrobials, AST interpretation most commonly employed human-specific CBPs (53.3%) or veterinary CBPs for another body site, organism or animal species (51.5%). Less commonly, laboratories would report no interpretation (30.9%) or test not possible/not performed (22.4%). Alternatively, they would use epidemiological cut-offs (ECOFFs) (21.2%) (Supplementary Table 4).

**FIGURE 4:**
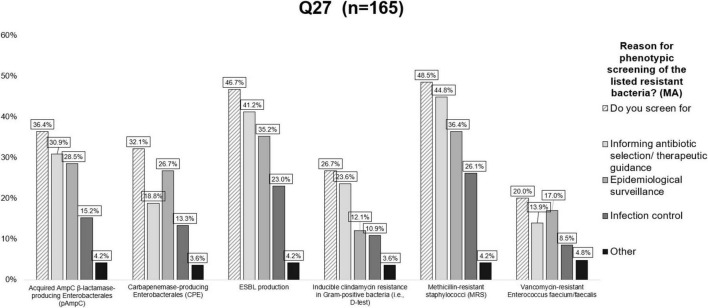
Responses to question Q27 on reasons for phenotypical screening of various resistant bacteria. For each type of resistant bacteria listed, multiple answers were possible. Valid answers were considered if participants interacted with at least one of the questions in a row of the table (see Supplementary Table 3). MA, multiple answers.

Most laboratories reported screening for MRSA/MRSP (48.5%) and ESBL-producing *Enterobacterales* (46.7%), followed by pAmpC (36.4%), carbapenemase-producing *Enterobacterales* (CPE) (32.1%), inducible clindamycin resistance in Gram-positive bacteria (26.7%) and vancomycin-resistant *Enterococcus* (VRE) species (20.0%) (Figure 4 and Supplementary Table 6). Detection of these resistance mechanisms was mostly performed by DD followed by MIC and molecular testing. The use of chromogenic media and sending isolates to reference laboratories were used to a lesser extent (Supplementary Table 5). Screening for ESBL production, pAmpC, methicillin resistance and inducible clindamycin resistance was reported as mostly performed for therapeutic guidance, whereas screening for CPE and vancomycin resistance in *Enterococcus* species was primarily performed for epidemiological surveillance (Figure 4 and Supplementary Table 6).

### Section C. Interpretation of results and reporting

To determine the clinical significance of bacterial isolates obtained upon culture, most laboratories (59.2%) reported always using knowledge on the most common organisms known to cause infections at the sampling site concerned. This was followed by knowledge of sampling method/site (56.7%), the duration/mode of sample transport (39.5%) and identification of the organism at species level (47.8%). Cytology reports and Gram-stained smears were less often used with 32.5% and 15.9%, respectively, of respondents claiming to never use these options (Supplementary Table 7).

The laboratories use different approaches for selection of isolates for AST, depending on whether cultures originate from normally sterile or non-sterile body sites (Supplementary Table 8). The most remarkable difference was that 31.2% of respondents selected all phenotypically distinct isolates for AST from cultures of normally sterile sites, whereas only 4.5% of respondents would do the same from non-sterile body sites.

Concerning reporting to clients, less than half of respondents generally add comments in their diagnostic answers to indicate if obtained bacterial isolates can be regarded as clinically significant (48.4%), likely commensal/resident flora (41.4%) or likely opportunistic bacteria (30.6%) (Supplementary Table 9). Two questions (Q31 and Q32) concerned the provision of information to clients concerning AST result and measures to promote prudent antimicrobial use. There was a tendency to rarely provide such information, although selective reporting of antimicrobials (i.e. excluding highest priority critically important antimicrobials from AST reports) was fairly common with 39.5% of respondents claiming to do that (Supplementary Tables 10, 11).

The survey revealed variety of approaches in reporting AST results, where most laboratories (47.9%) only included the qualitative interpretation of the result (S, R, and I), whilst others provided the actual MIC value with (25.3%) or without (36.5%) displaying the CBPs used for interpretation (Supplementary Table 12). Moreover, a couple of laboratories took a more complex approach, e.g., by indicating the ratio between the CBP and the MIC.

### Section D. Surveillance, laboratory data management and further developments

The survey identified that most laboratories had a data management system for sample recording (86.1%) and reporting (86.0%). Additionally, the majority of respondents claimed to be able to archive and extract culture and AST results from this system (91.3%), and to extract data for analyzing AMR trends (77%) (Supplementary Table 13).

Many respondents indicated that they participated in pathogen or AMR surveillance programs. Almost 60% of laboratories participated in *Salmonella* reporting and 44.4% participated in broader zoonotic pathogen surveillance. Participation in AMR surveillance programs was generally poor, with a higher participation rate in farm animal (53.6%) compared to companion animal (40.1%) schemes. (Supplementary Table 14).

Overall, laboratories strongly supported the development of specific guidelines, especially guidelines for interpreting and reporting of AST results (Supplementary Table 15).

## Discussion

The survey identified broad variability in practices between laboratories, underscoring the well-recognized problem of veterinary diagnostic laboratories not adopting uniform microbiological procedures (Guardabassi et al., 2017; Timofte et al., 2021). In the survey, this shortcoming in the veterinary microbiology laboratories practices was observed both within a country (if more than one laboratory answered the survey) and between countries, adding to the conclusion that the lack of standardized microbiology practices is a general problem that needs to be addressed. These differences can be attributed to various factors including training background, lack of broad consensus for the use of a common methodology in veterinary laboratories with multiple standards (e.g. EUCAST, CLSI or national committees) adopted depending on local factors and choices, access to resources and new technologies, reporting methodology etc.

Besides the lack of inharmonious methodology, the survey identified diverse training backgrounds and expertise of the microbiology diagnostic teams. Although our survey did not address the issue of how “veterinary microbiologist” training was achieved, only 55.7% of laboratories reported to have a head of service/director with a primarily veterinary microbiology-based background. This seems to be a wider issue, which is also affecting human clinical microbiology (Humphreys et al., 2010). Interestingly, a recent US-based publication has highlighted the need for well-trained and qualified medical microbiologists as directors of clinical microbiology laboratories (Samuel et al., 2021). In addition, the Infectious Disease Society of America (IDSA) guideline is recognizing the value of medical microbiologists as core members of any antimicrobial stewardship programme, and their potential to significantly impact antibiotic usage (Dellit et al., 2007). The establishment of the European College of Veterinary Microbiology (ECVM) in 2016 promoting standardized training and recognition of the professional identity of veterinary microbiologists will help building specific expert capacity for veterinary microbiology laboratories in the future.

Laboratory performance can also be impacted by financial capacity or willingness to invest in technologies, which can greatly facilitate faster and more accurate results. In that regard it is worth noting that whilst 77% of responding laboratories still used individual biochemical identification assays for various reasons, 43.3% of laboratories reported having access to a MALDI-TOF MS for bacterial/fungal species identification. This is encouraging, as MALDI-TOF has revolutionized clinical microbiology by greatly improving the turnaround time and accuracy of bacterial and fungal identification at species level (Patel, 2015; Van Driessche et al., 2019). The rapid and accurate identification offered by MALDI-TOF significantly impacts how bacterial cultures are interpreted and which isolates are selected for AST, as laboratories need to ensure that their bacterial culture and AST results are clinically relevant. Since MALDI-TOF equipment constitutes a big economic investment, use of conventional phenotypic tests is unlikely to be replaced in small laboratories and/or limited resource settings. On the other hand, MALDI-TOF is more cost-effective for large clinical microbiology laboratories due to its low cost per sample, reduced reagent costs, labor, and turnaround time, as described by several studies (Bizzini and Greub, 2010; Thompson, 2022; Calderaro and Chezzi, 2024).

Bacterial culture interpretation is a valuable and complex skill, which makes use of a plethora of clinical and paraclinical information to identify isolates likely associated with an infection. In this context, the survey showed that, for instance, knowledge of organisms likely to be aetiological agents at the infection site was commonly taken into consideration (59.2%). The use of Gram-stained smears from clinical specimens has been shown to be a key step in interpretation of bacterial culture results as well as for guiding empiric antimicrobial therapy (Musher et al., 2004; Stone and Steele, 2009). However, it is surprising that Gram-stained smear findings or evidence of inflammation from cytology reports was an identification criterion used consistently by only 24.8% and 15% of labs, respectively. This may reflect lack of expertise for smear staining or interpretation, laboratories being too busy or not having enough personnel resources for these informative, but time-consuming evaluation tools. This finding is related to another important issue concerning selection of clinically relevant isolates for AST: our data showed that 23.4% of laboratories claimed to always select only pure growth isolates from non-sterile body sites (e.g., skin, mucosal surfaces) for AST, whilst 62% of laboratories would select up to two or three isolates. Such large variation between laboratories reflects that there is not yet international consensus on how to select isolates from such samples, which may indeed be difficult and should also rely on other factors such as the relative proportion of colonies and sample type and origin. Nevertheless, this result suggests that training and guidance in this area is acutely needed, as isolate selection for AST is a key element directly impacting antibiotic use. Remarkably, whilst several standards describe how to perform AST (CLSI, EUCAST, WHO, etc.) (OIE, 2020; CLSI VET01, 2024; CLSI VET01S, 2024 EUCAST, 2024), there is little guidance on which isolates to select for AST. It is therefore encouraging that some recently published training resources from human medicine (CDC, 2020), are more specific about the need for laboratory procedures to reflect best practices for the workup of clinical specimens and emphazising the importance of isolate selection for AST. Guidelines in this area could reduce the risk of performing AST on commensal or contaminating organisms (CDC, 2020). To this end, ENOVAT organized two consecutive training schools focused on bacterial culture interpretation of veterinary clinical specimens and isolate selection for AST.

Maybe unsurprisingly, the survey also showed that communication between the laboratory and clinicians was rare (22.3% only). This might reflect the distancing of clinical microbiology services from the patient, as is the case for most commercial laboratory settings. We do however encourage regular communication between the laboratory and clinician, since requisition schemes often lack important patient- or sample-related information that could be used to guide the approach taken in the laboratory. Communication is also valuable from the perspective of the clinician who may benefit from help with interpretation of AST results, and consequently with selection of appropriate treatment.

One of the most important findings of this study was the identification of combined approaches being used for interpreting AST results, with a notable proportion of responding laboratories using a combination of CLSI, EUCAST (47.6% for MIC and 41.2% for DD) and national guidelines (16.9% for DD and 6.7% for MIC) for interpretation of results. On the contrary, few respondents (as low as 15.2% for MIC and 17.6% for DD) used either EUCAST or CLSI guidelines exclusively. The combination of multiple standards for interpreting AST in veterinary medicine can be considered a necessity, as CBPs are missing for several antimicrobial agent−animal species−body site−pathogen combinations. This is also reflected in our finding that 51.5% and 53.3% of laboratories reported the use of CBPs developed for other body sites/organisms/animal species or for humans, respectively, when a specific CBP is missing. While realizing that the shortage of specific CBPs cannot be solved in the short term, a guideline on how to prioritize among non-specific CBPs has been recently developed by CLSI (CLSI VET09, 2024). Despite providing useful support, this guideline also emphasizes that the clinical validity of non-specific CBPs is often questionable. One additional downside of using many different interpretive criteria for AST is the complexity of comparing AMR data across laboratories unless raw data (MICs or inhibition zone diameters) are provided. In that regard, several recent studies have analysed the discrepancies in results interpretation when applying either CLSI or EUCAST CBPs to various pathogen-antimicrobial combinations, supporting the claim for a globally harmonized AST system that would be both practical and freely available (Delgado-Valverde et al., 2017; Cusack et al., 2019; EFSA Panel on Animal Health and Welfare [AHAW] et al., 2021; Maganga et al., 2023). At the same time, there are profound differences in the ways CLSI and EUCAST are set up and governed which can generate practical differences between their methodologies and interpretative criteria, making the systematic harmonization of the two committees challenging (Kahlmeter, 2015).

Reporting AST results effectively is crucial for guiding clinical decision-making and promoting antimicrobial stewardship. In that context the laboratories can employ a number of approaches such as selective and cascade reporting of AST results (Guardabassi et al., 2017). The survey results demonstrated that selective reporting of antimicrobials was the most common approach taken by the laboratories when reporting AST results (39.5%), meaning not reporting the results for the highest priority critically important antimicrobials. However, 37.6% of laboratories do not take any specific approach when reporting AST results. Therefore, it’s crucial to encourage practices in AST reporting that align with the principles of antimicrobial stewardship, so the clinicians can be directed towards prescribing narrower-spectrum or first-line antibiotics. This strategy aids in conserving broader-spectrum antibiotics and those critical for human health, reserving them for instances where they are indispensable.

The determination of MIC of an antimicrobial agent offers valuable information in terms of drug choice, dose and frequency of treatment. The survey results indicated that the laboratories using MIC-based tests have a variety of strategies when reporting i.e. reporting only the susceptibility, both susceptibility and the MIC value, as well as susceptibility, MIC value and the breakpoints values. More detailed and comprehensive information reported by the veterinary laboratories like the breakpoint-MIC ratio is important in guiding the drug selection since the distance of the MIC from the breakpoint is relevant for the level of susceptibility of the pathogen to the selected antimicrobial. Additionally, the selection of an antibiotic should not solely depend on the MIC value. It is important to consider a variety of factors including the distance of the MIC from the breakpoint, infection site, the species, age and health status of the animal, drug’s pharmacokinetics, as well as the route and frequency of administration. Due to factors like pharmacokinetics and poor tissue penetration, which can result in subtherapeutic drug levels at the site of infection, using an antimicrobial with a lower MIC relative to the breakpoint is recommended. Additionally, for drugs with an MIC near the breakpoint, increasing the dosage or frequency of administration may be necessary to achieve sufficient drug levels at the infection site reducing the risk of treatment failure (Allerton and Nuttall, 2021). In this context, guidance to the clinicians is crucial in the interpretation of the MIC test results, which will facilitate appropriate antibiotic selection, improve treatment outcome, promote antimicrobial stewardship and ultimately tackle AMR.

The lack of specific guidelines for detection of AMR mechanisms is reflected by the plethora of responses when laboratories were asked if, and for what reason, they screened bacterial isolates for resistance mechanisms (e.g., epidemiological surveillance, informing antibiotic therapy or infection control). In this context, it is remarkable that only 48.5% of laboratories screened for methicillin resistance (MR), and only 26.7% also screened for inducible clindamycin resistance in staphylococci. Even fewer labs screened for CPE and VRE. This is probably due to the fact that these antimicrobial classes (carbapenems and glycopeptides) are not registered for use in animals meaning that for therapeutic guidance this screening is not needed. However, for epidemiological surveillance it would be very interesting to screen for these primarily human-relevant resistance mechanisms in the scope of One Health. Surveillance of such resistance types was also recommended by the European Antimicrobial Resistance Surveillance Network (EARS-Vet), even though the primary focus of this network is AMR in clinical isolates of animal origin (Mader et al., 2022a).

Survey respondents clearly indicated their desire to have access to common guidelines for bacterial culture, isolation and identification, and to be recommended preferred guidelines to follow for interpretation of AST. Consequently, and unsurprisingly, there is a need for standardization of the bacteriological diagnostic process from sample collection, processing, pathogen identification, selection of isolates for AST, and reporting in laboratories across all veterinary diagnostic providers. Some national initiatives, such as RESAPATH (the French network for surveillance of AMR in bacteria from diseased animals, available at),^3^ offer an example of achieving common methodology for national harmonization of AMR surveillance. At European level, work led by the EARS-Vet framework mapped national monitoring systems for AMR in bacterial pathogens of animals (both companion and food-producing) among 27 countries, reviewing their structure and operations and generating useful information for countries planning to build or improve their AMR systems (Mader et al., 2022b). These authors showed important gaps in the current landscape of AMR surveillance in animals, and they proposed a pragmatic AST harmonized strategy. Similarly, data accumulated via the current survey highlights gaps to be addressed for optimizing and harmonizing veterinary diagnostic laboratory practices and will serve as the foundation for tackling the main gaps identified. As such, a subgroup of ENOVAT members is working towards building an archive of Veterinary Microbiology Protocols (initially focusing on companion animal clinical specimens) as the first steppingstone towards achieving the long-held goals of harmonization of bacteriological diagnostic procedures across veterinary microbiology laboratories in Europe and beyond.

The findings from this study have some limitations. First, due to the extent of the ENOVAT Network (> 300 members from > 40 countries) who were asked to disseminate the link to the survey, we cannot know exactly how many laboratories were reached and invited to participate. This means that we cannot be certain that the findings represent the laboratory approaches to bacterial culture and AST in all these countries. Another limitation is that there was a large variation in laboratory settings making it hard to compare responses. For instance, governmental laboratories mainly focus on screening for specific zoonotic pathogens or resistance phenotypes in defined sample types from farm animals (e.g., faeces), whereas commercial laboratories may receive a large variety of clinical samples from companion animals. Additionally, data from the survey was not adjusted for the number of laboratories in each participating country and other country-specific metrics, which does not allow for comparisons between countries and country-specific conclusions. As accurate information on the exact number of practising laboratories in each participating country was not available, future studies looking to capture these data and using country-specific metrics coupled with statistical analysis will be beneficial to draw unequivocal country-specific conclusions.

## Conclusion

We identified a broad variety of methodologies being used for bacterial culture and AST in European veterinary microbiological diagnostic laboratories. Although some responses (e.g., failure to identify bacteria to species level) are against the general perception of good diagnostics, the overall variation in responses was expected, since internationally acknowledged veterinary-specific guidelines are lacking for many steps of bacterial culture beyond AST (from sample receipt to reporting).

The diversity in methodologies for C&ID, lack of consensus on isolate selection for AST, combined use of multiple guidelines for interpreting AST results and variation in AST reporting practices emphasize the need for an internationally harmonized approach in veterinary clinical microbiology. Furthermore, the inconsistent screening for AMR mechanisms requires development of standardized protocols, especially for epidemiological surveillance in a One Health context.

We therefore call for the development of specific guidelines and standards for processing clinical specimens to support veterinary laboratories. Furthermore, resources need to be dedicated to ensuring that laboratory staff are trained appropriately, and technical facilities are available to support them. Clinical staff also need to be trained to interpret the results and to communicate regularly with laboratories, thereby ensuring the best foundation for diagnostic-driven antimicrobial stewardship.

## Data Availability

The original contributions presented in this study are included in this article, further inquiries can be directed to the corresponding author.

## Footnotes

1
www.ecvmicro.org

2
https://enovat.eu/

3
https://resapath.anses.fr/
