# Supplementary material for: Towards harmonized laboratory methodologies in veterinary clinical bacteriology: outcomes of a European survey
Source: Front Microbiol. 2024 Oct 10;15:1443755. doi: 10.3389/fmicb.2024.1443755 (PMC11499178; doi:10.3389/fmicb.2024.1443755)
Supplement: Supplementary file 1 [file Data_Sheet_1.pdf]

Towards harmonized laboratory methodologies in veterinary clinical bacteriology: outcomes of a European survey

Tom Koritnik[1]†, Iskra Cvetkovikj[2]†, Flavia Zendri[3], Shlomo Eduardo Blum[4], Serafeim Christos Chaintoutis[5], Peter A. Kopp[6], Cassia Hare[7], Zrinka Štritof[8], Sonja Kittl[9], José Gonçalves[10], Irena Zdovc[11], Erik Paulshus[12], Andrea Laconi[13], David Singleton[3], Fergus Allerton[14], Els M. Broens[15], Peter Damborg[16] and Dorina Timofte[3]*

†These authors contributed equally

Supplementary Material

Supplementary Data

Supplementary Materials include (1) questions (n=37) from the survey disseminated to veterinary diagnostic laboratories across Europe and (2) supplementary tables (n=15) providing an overview of responses to questions in the survey.

1) **Survey**

ENOVAT WG1-Diagnostic procedures survey

1. Participant Information

The Diagnostic Procedures Survey – is designed to get an insight into methodology and standards used by veterinary microbiological laboratories across Europe.

If you decide to take part and complete the online survey, this will take around 20-25 minutes.

Before you decide whether to participate, it is important for you to understand why this survey is being conducted and what it involves. Please take a few minutes to read the information provided below.

Why am I being invited to take part?

This survey is part of the "European Network for Optimization of Veterinary Antimicrobial Treatment" (ENOVAT) project funded by the EU COST Action in which 32 countries are taking part. More information and the list of participating countries can be found at: https://enovat.eu/

The aim of this Action is to optimize veterinary antimicrobial use with special emphasis on the development of antimicrobial treatment guidelines and refinement of microbiological diagnostic procedures. For this purpose, this survey will primarily investigate the methods and interpretive criteria used by veterinary diagnostic laboratories across Europe for a) pathogen identification and b) antimicrobial susceptibility testing (AST), as well as c) the approaches taken for supporting appropriate antimicrobial selection by clinicians.

Your participation in the survey will increase our chances of acheiving the goals of this project, which overall aims to improve the quality of veterinary diagnostics, treatment guidelines and improve veterinary antimicrobial stewardship.

Do I have to take part?

Participation is voluntary and you do not have to take part in this study. If you feel that someone else in your organization is better placed to complete the survey, then please pass this on.

What will happen if I want to stop taking part?

You can withdraw at any time during the online survey. However, once the questionnaire is completed and submitted, we will not be to withdraw your information because the survey is run anonymously and we cannot identify your responses.

How will my data be used?

The data collected will be used only for this specific project and only a limited number of people will have access to the data. The data you provide will be stored securely on the University of Liverpool password protected computers for 7 years, in line with data protection requirements at the University of Liverpool.

What will happen to the results of the survey?

The results from this survey will be used to identify the current state-of-play in veterinary microbiology diagnostics methodologies across Europe, with focus on methodologies and interpretive criteria used for bacterial identification and AST of veterinary pathogens. Once the data is collected and analysed, we aim to present the results in an open access journal and through the relevant veterinary press.

If you have any questions about the survey, please contact:

Dorina Timofte

d.timofte@liv.ac.uk

University of Liverpool

Leahurst Campus

Chester High Road

CH64 7TE, UK

If we cannot answer your question or you have a complaint with which you feel you cannot come to us, then please contact the University of Liverpool Research Ethics and Integrity Office at ethics@liv.ac.uk

Please quote the study name ENOVAT, the researcher involved, and the details of the complaint you wish to make.

* 1. Please confirm that you have read and understood the above information and consent to participating in this survey:

◯ Yes, I have read the above information and I consent to participation in this study

◯ No, I do not consent to participation in this study

ENOVAT WG1-Diagnostic procedures survey

2. Section A.

About your laboratory (Q1-17)

* 2. In what **country** do you work?

* 3. Does your laboratory offer bacterial culture and susceptibility testing?

◯ Yes

◯ No

ENOVAT WG1-Diagnostic procedures survey

3. Section A.

About your laboratory (Q1-17)

* 4. What is the **main sector (setting)** in which your laboratory is functioning (please select ONE option):

- ◯ Academic
- ◯ Private
- ◯ Governmental
- ◯ Charity; Non-governmental organisation
- ◯ Other (please specify)

ENOVAT WG1-Diagnostic procedures survey

4. Section A.

About your laboratory (Q1-17)

* 5. Specify the private sector that you work in (please select ONE option):

- ◯ Commercial laboratory
- ◯ In house veterinary practice/hospital laboratory
- ◯ Industry
- ◯ Private Research Institute
- ◯ Other (please specify)

* 6. Does your laboratory give **guidance** for optimal specimen collection and management:

- ◯ Yes
- ◯ No

ENOVAT WG1-Diagnostic procedures survey

5. Section A.

About your laboratory (Q1-17)

* 7. How do you provide this guidance (select ALL that apply)?

- [ ] Website
- [ ] Submission forms
- [ ] Email
- [ ] Telephone
- [ ] Other (please specify)

* 8. What aspects of specimen collection and management are covered in your guidance (select ALL that apply)?

- [ ] Sampling site
- [ ] Sampling techniques for different sample types
- [ ] Devices/tubes/systems used to collect different sample types
- [ ] Packaging of clinical samples
- [ ] Storage of clinical samples
- [ ] Timing of collection (for example, in relation to antimicrobial treatment)
- [ ] Other (please specify)

* 9. What is the **main specimen type** that you process in your laboratory (Please select all those that apply).

- [ ] Clinical
- [ ] Foodstuff and animal feed
- [ ] Environmental

Other (please specify)

ENOVAT WG1-Diagnostic procedures survey
6. Section A.
About your laboratory (Q1-17)

* 10. What is the **total number of clinical specimens** that are processed for bacteriology by your laboratory each year (please select ONE option).

- ◯ < 3000/year
- ◯ 3000-4999/year
- ◯ 5000-7999/year
- ◯ 8000-12000/year
- ◯ > 12000/year

* 11. From **what animal species** do you process clinical specimens for culture and antimicrobial susceptibility testing.

- ☐ Farm animals (e.g. cattle, small ruminants, pigs, farmed rabbits, mink and/or poultry)
- ☐ Small animals (dogs and/or cats)
- ☐ Equine
- ☐ Exotic pets (e.g. pet birds, rabbits, rodents, reptiles and/or ornamental fish)
- ☐ Wildlife/Zoo animals
- ☐ Laboratory animals
- ☐ Fish

* 12. Does your laboratory have an **Internal Quality Assurance (QA)?** (as defined by WHO "the total process whereby the quality of laboratory reports can be guaranteed")

- ◯ Yes
- ◯ No

ENOVAT WG1-Diagnostic procedures survey

7. Section A.

About your laboratory (Q1-17)

* 13. Please give details of which type of**Internal Quality Assurance** is implemented in your laboratory (tick as appropriate)

- [ ] Good laboratory practice manual
- [ ] Development programs (or competency assessment) for staff
- [ ] Standard Operating Procedures
- [ ] Equipment maintenance and calibration
- [ ] Use of Quality Control strains
- [ ] Other (please specify)

* 14. Does your laboratory have an External Quality Assurance?

- ( ) Yes
- ( ) No

ENOVAT WG1-Diagnostic procedures survey

8. Section A.

About your laboratory (Q1-17)

* 15. Please give details of which type of**External Quality Assurance** is implemented in your laboratory (tick as appropriate)

- ☐ External audits
- ☐ Taking part in national proficiency testing
- ☐ Taking part in a European proficiency testing scheme (i.e, EU Reference Laboratory for Antimicrobial Resistance)
- ☐ Accreditation from a recognised QA system (e.g, ISO)
- ☐ Other (please specify)

* 16. The **Microbiology Diagnostics Laboratory team** in your laboratory includes (Please select all those that apply):

- ☐ Technical staff
- ☐ Veterinary microbiologist(s)
- ☐ Microbiologist(s) (non-veterinary background)
- ☐ Veterinary clinical pathologist(s)
- ☐ Veterinary pathologist(s)
- ☐ Veterinary internal medicine specialist(s)
- ☐ Veterinary nurse
- ☐ Veterinarian/clinician
- ☐ Other (please specify)

* 17. What is the professional background (or training level) of the **Head of Laboratory or Laboratory Director**:

- Technical staff
- Veterinary microbiologist
- Microbiologist (non-veterinary background)
- Veterinary clinical pathologist
- Veterinary pathologist
- Veterinary internal medicine specialist
- Veterinary nurse
- Veterinarian/clinician
- Other (please specify)

* 18. What is the **average turnaround time** for culture and antimicrobial susceptibility testing results of fast growing organisms, e.g. *Escherichia coli* (tick as appropriate).

	1-2 days	3-5 days	6-8 days	Other
Bacterial identification				
Antimicrobial susceptibility testing				

Other (please specify)

ENOVAT WG1-Diagnostic procedures survey

9. Section B.

Methodology (bacterial culture, identification and susceptibility testing) (Q18-26)

* 19. Does your **laboratory provide** (select ALL those that apply):

- [ ] Aerobic culture
- [ ] Anaerobic culture
- [ ] Microaerophilic culture, e.g. *Campylobacter* spp
- [ ] Culture for organisms growing in 5-10% CO2, e.g. *Haemophilus* spp
- [ ] Mycobacterial culture
- [ ] Mycoplasma culture
- [ ] Selective culture of target organisms (please specify below)
- [ ] Other (please specify)

* 20. Do you attempt to **identify bacterial isolates** at species level? (select ONE option)?

- ( ) Yes
- ( ) In most cases (>50% of cases)
- ( ) In some cases (<50%)
- ( ) No

* 21. What methods/systems, do you use for **bacterial identification?** (select all that apply):

- [ ] Individual biochemical tests (e.g. catalase, oxidase)
- [ ] API kits (Biomerieux or similar)
- [ ] Vitek-1 or Vitek-2
- [ ] Trek SENSITITRE
- [ ] MALDI-TOF MS
- [ ] MicroScan Walkaway
- [ ] BD Phoenix
- [ ] PCR (16s rRNA sequencing or other gene target)
- [ ] Other (please specify)

* 22. Routinely, do you provide **antimicrobial susceptibility testing** by (Please select all that apply):

- [ ] Minimum Inhibitory concentrations (MIC), by default
- [ ] MIC by request
- [ ] Disc diffusion (Kirby-Bauer) by default and MIC by request
- [ ] Disc diffusion (Kirby-Bauer) by default
- [ ] Disc diffusion by request

ENOVAT WG1-Diagnostic procedures survey

10. Section B.

Methodology (bacterial culture, identification and susceptibility testing) (Q 18-27)

23. What method do you use for **MIC antimicrobial susceptibility testing** (select all that apply)?

- [ ] Vitek-1 or Vitek-2
- [ ] Trek SENSITITRE
- [ ] MicroScan Walkaway
- [ ] BD Phoenix
- [ ] Micronaut AST system
- [ ] In house broth microdilution
- [ ] In house agar dilution
- [ ] Gradient test strip (e.g. E-test)
- [ ] Other (please specify)

* 24. Which are the main clinical breakpoints you use for interpretation of antimicrobial susceptibility testing results (disc-diffusion and MIC)?

	EUCAST	CLSI	Combination of EUCAST/CLSI	National guidelines	Other
Disc diffusion (Kirby-Bauer)	○	○	○	○	○
MIC	○	○	○	○	○

Other (please specify)

*** 25. What approach do you take for interpretation of antimicrobial susceptibility testing (AST) when there are no animal/organ/pathogen-specific clinical breakpoints (CBPs) for one or more antimicrobials?**

- [ ] Use a CBPs from another organ/organism/animal species for which there are published veterinary CBPs;
- [ ] Use CBPs developed for infection in humans;
- [ ] Use epidemiological cutoffs (ECOFFs)* instead of CBPs;
  ** Epidemiologic cut off values, which separate bacterial populations into wild type and those with acquired or mutational resistance to a particular antimicrobial agent.*
- [ ] Report that the isolated organism cannot be tested due to the lack of CBPs required for interpretation of AST;
- [ ] Testing but not interpreting antimicrobial susceptibility results and include a comment to guide treatment choice, e.g "is likely to be susceptible to….." or "likely to be resistant to…."

Other (please specify)

* 26. What methods do you use for **detection and interpretation of Antimicrobial Resistance (AMR)** phenotypes? Also, please specify if you refer AMR isolates to any Reference Laboratories for verification (tick as appropriate)?

	Disc detection	MIC testing	Chromogenic media	Molecular detection (e.g., PCR or PBP2 agglutination test for MRSA/MRSP)	Send isolates to reference labs	N/A	Other
MRSA/MRSP	☐	☐	☐	☐	☐	☐	☐
ESBL-producing Enterobacteriaceae	☐	☐	☐	☐	☐	☐	☐
Vancomycin resistant *Enterococcus faecium/faecalis*	☐	☐	☐	☐	☐	☐	☐
Inducible clindamycin resistance in Gram-positive bacteria	☐	☐	☐	☐	☐	☐	☐
Acquired AmpC β-lactamase-producing Enterobacteriaceae (pAmpC)	☐	☐	☐	☐	☐	☐	☐
Polymyxin resistance in Gram-negative bacilli	☐	☐	☐	☐	☐	☐	☐
Carbapenemase-producing Enterobacteriaceae (CPE)	☐	☐	☐	☐	☐	☐	☐

Other (please specify)

* 27. In your laboratory (i) **do you screen** phenotypically for the listed resistant bacteria (ii) for any of the reasons shown? (tick as appropriate)

	Do you screen for	Informing antibiotic selection/ therapeutic guidance	Epidemiological surveillance	Infection control	N/A	Other
ESBL production	☐	☐	☐	☐	☐	☐
Acquired AmpC β-lactamase-producing Enterobacteriaceae (pAmpC)	☐	☐	☐	☐	☐	☐
Carbapenemase-producing Enterobacteriaceae (CPE)	☐	☐	☐	☐	☐	☐
Methicillin-resistant staphylococci (MRS)	☐	☐	☐	☐	☐	☐
Vancomycin-resistant Enterococcus faecium/faecalis	☐	☐	☐	☐	☐	☐
Inducible clindamycin resistance in Gram-positive bacteria (i.e., D-test)	☐	☐	☐	☐	☐	☐

Other (please specify)

ENOVAT WG1-Diagnostic procedures survey
11. *Section C.*
Results interpretation and reporting (Q28-34)

* 28. Are **bacterial culture reports** accompanied by **comments** regarding the clinical significance of the isolated organisms by providing an estimate on their likely role, as follows? (tick as appropriate):

	Yes	No	On "ad hoc" basis	Other
Likely commensal/resident flora	◯	◯	◯	◯
Likely opportunistic pathogenic	◯	◯	◯	◯
Clinically significant	◯	◯	◯	◯

Other (please specify)

* 29. When **assessing clinical significance** of bacterial isolates obtained from clinical specimens, what information – when available - is used routinely to make this decision? (tick as appropriate)

	Always	Often (60-99% of cases)	Sometimes (30-60% of cases)	Rarely (1-30% of cases)	Never
Knowledge of sampling method/sampling site	◯	◯	◯	◯	◯
Duration/mode of sample transport	◯	◯	◯	◯	◯
Clinical history provided on the submission form	◯	◯	◯	◯	◯
Specimen Gram-stained smear findings	◯	◯	◯	◯	◯

	Always	Often (60-99% of cases)	Sometimes (30-60% of cases)	Rarely (1-30% of cases)	Never
Evidence of inflammation from cytology report (from clinical pathology)	◯	◯	◯	◯	◯
Knowledge of organisms likely to be etiologic agents at the infection site	◯	◯	◯	◯	◯
The amount of growth obtained	◯	◯	◯	◯	◯
The number and relative proportion of distinct organisms cultured	◯	◯	◯	◯	◯
Identification of the organisms at species level	◯	◯	◯	◯	◯
Information obtained from discussion with the clinician in charge of the case	◯	◯	◯	◯	◯
Other	◯	◯	◯	◯	◯

Other (please specify)

* 30. In your laboratory, **how many isolates do you typically select** for antimicrobial susceptibility testing from one sample? (tick as appropriate)

	All bacterial isolates representing all colony types	Pure growth isolates only	Up to max 3 isolates	Up to max 2 isolates	Other
Normally sterile body sites (e.g., blood, CSF, synovial fluid)	○	○	○	○	○
Non-sterile body sites (e.g.., skin, mucosal surfaces)	○	○	○	○	○

Other (please specify)

* 31. Do you as a default include the **following recommendations** when sending your culture and antimicrobial susceptibility results to the requesting veterinarian? (tick as appropriate)

	Always	Often (60-99%)	Sometimes (30-60%)	Rarely (1-30%)	Never
Indicate what antimicrobials are suitable for different infection types and/or body sites	○	○	○	○	○
Indicate suitable antimicrobials for organisms for which AST is difficult to perform (e.g., anaerobic organisms)	○	○	○	○	○
Indicate that for MRSA/MRSP/ESBL carriers, antibiotic treatment is not indicated	○	○	○	○	○
Show Breakpoints that were applied (e.g., CLSI-VET08, 2018)	○	○	○	○	○
Specify when results are “Not Interpretable” due to the lack of an agreed breakpoint for certain organisms/antimicrobials combination	○	○	○	○	○

* 32. When **reporting antimicrobial susceptibility testing results**, what approach do you generally take to encourage prudent antimicrobial use? (tick as appropriate)

- ☐ **Selective reporting** [I.e. , suppress Highest Priority Critically Important Antimicrobials (HPCIAs) results which may be included in commercial animal panels (i.e, carbapenem, vancomycin, and linezolid)]
- ☐ **Cascade reporting** (i.e., report AST results for only one drug within a certain class, if Susceptible to both (i.e., report gentamicin but not amikacin)
- ☐ **Group antimicrobials by "tier"** OR 1st line, 2nd line, etc.; with a comment indicating that higher tier should only be used on organisms resistant to 1st line
- ☐ Indicate which a**ntimicrobials are suitable according to the site of infection** and antibiotic penetration
- ☐ **Indicate** when topical and not systemic usage would be appropriate
- ☐ **Indicate** "Doubtful clinical significance, no treatment is indicated"
- ☐ **Indicate** "Discussion with clinician required? "
- ☐ No **specific approach** taken
- ☐ Other (please specify)

*** 33. If your laboratory performs MIC for antimicrobial susceptibility testing, what specific information do you provide on the results report?**

	Always	Upon request	N/A
The clinical interpretation category (**S, R, I**) only (**without MIC values**)	◯	◯	◯
The clinical interpretation category (**S, R, I**) and **the MIC value** (µg/ml) **without breakpoints** used for interpretation (e.g. if you use TREK SENSITITRE)	◯	◯	◯
The clinical interpretation category (**S, R, I**) and the MIC value (µg/ml) **with the breakpoint values** used for interpretation	◯	◯	◯
Other	◯	◯	◯

Other (please specify)

* 34. If your laboratory provides MIC values, do you provide any **guidance to clinicians on how to use the MIC values** for antibiotic selection? (Please select one)

- ◯ Yes
- ◯ No
- ◯ On request
- ◯ N/A

ENOVAT WG1-Diagnostic procedures survey

12. Section D.

Surveillance, laboratory data management and further developments (Q35-37)

* 35. Please indicate what **data management system** do you use in your laboratory (tick as appropriate):

	Yes	No	N/A
Do you use a computerised system for sample recording/accessioning?	○	○	○
Do you use a computerised system for reporting?	○	○	○
Are you able to store and extract culture and susceptibility testing results?	○	○	○
Can you extract data to analyse antimicrobial resistance trends?	○	○	○

* 36. Do you take part in any **pathogen surveillance schemes** (tick as appropriate)?

	Yes	No
Antimicrobial resistance, for farm species	○	○
Antimicrobial resistance, for companion animals	○	○
Salmonella reporting and typing	○	○
Zoonotic pathogens surveillance	○	○
Other	○	○

Other (please specify)

* 37. If new specific **guidelines for veterinary clinical microbiology laboratories** are developed, please score which you consider the most important aspects that will make a difference in the quality of the results you provide? (where 1 = not important and 5= highly important):

	1	2	3	4	5
Provide common guidelines for bacterial culture, isolation and identification	○	○	○	○	○
Issue guidelines for interpreting and reporting clinical significance of bacterial cultures	○	○	○	○	○

	1	2	3	4	5
Recommend “preferred” guidelines for interpreting and reporting antimicrobial susceptibility results (CLSI-VAST or EUCAST)	◯	◯	◯	◯	◯
Issue guidelines for what to do in the absence of breakpoints	◯	◯	◯	◯	◯
Issue guidelines for surveillance of antimicrobial resistance in veterinary isolates	◯	◯	◯	◯	◯
Provide Expert rules on intrinsic resistance and exceptional phenotypes for veterinary pathogens	◯	◯	◯	◯	◯
Provide guideline for the detection of resistance mechanisms including specific resistances of clinical and/or epidemiological importance	◯	◯	◯	◯	◯

Other (please specify)

1) **Supplementary Tables**

Table S 1. *Responses on country of participants.*

		Number	Percentage
**Q2**	**In what country do you work? (SA)**	**n= 241**	
	UK	27	11.2%
	France	26	10.8%
	Croatia	15	6.2%
	Italy	15	6.2%
	Turkey	14	5.8%
	Germany	13	5.4%
	Romania	11	4.6%
	Greece	10	4.1%
	Poland	10	4.1%
	Belgium	9	3.7%
	Portugal	8	3.3%
	Norway	7	2.9%
	Moldova	6	2.5%
	Switzerland	6	2.5%
	Finland	5	2.1%
	Georgia	5	2.1%
	Ireland	5	2.1%
	Morocco	5	2.1%
	Serbia	5	2.1%
	Denmark	4	1.7%
	Israel	4	1.7%
	Netherlands	4	1.7%
	Spain	4	1.7%
	Austria	3	1.2%
	Bosnia and Herzegovina	3	1.2%
	Slovakia	3	1.2%
	Sweden	3	1.2%
	Ukraine	3	1.2%
	Lithuania	2	0.8%
	Slovenia	2	0.8%
	Albania	1	0.4%
	Bulgaria	1	0.4%
	Estonia	1	0.4%
	North Macedonia	1	0.4%

Table S 2. *Information provided about laboratories of respondents. Percentages are based on the number of responses to individual questions. MA, multiple answer question; SA, single answer question; IQA, internal quality assurance; EQA, external quality assurance.*

		**Number**	Percentage
**Q4**	**Main sector of operation (SA)**	n= 234	
	Academic	88	37.6%
	Governmental	64	27.4%
	Private	62	26.5%
	Other (mixture of two other types)	15	6.4.%
	Charity; Non-governmental organisation	5	2.1.%
**Q5**	**Private sector type (SA)**	n= 61	
	Commercial laboratory	40	65.6%
	In house veterinary practice/hospital laboratory	16	26.2%
	Industry	2	3.3%
	Other (please specify)	2	3.3%
	Private Research Institute	1	1.6%
**Q10**	**Number of samples processed (SA)**	n= 190	
	< 3000/year	110	57.9%
	> 12000/year	30	15.8%
	3000-4999/year	27	14.2%
	5000-7999/year	14	7.4%
	8000-12000/year	9	4.7%
**Q6**	**Guidance offered for collection and management (SA)**	n= 229	
	Yes	172	75.1%
	No	57	24.9%
**Q7**	**How do you provide guidance (MA)**	n= 159	
	Telephone	115	72.3%
	Email	91	57.2%
	Website	75	47.2%
	Submission forms	66	41.5%
	Other (specified)	33	20.8%
**Q8**	**Aspects covered in the guidance (MA)**	n= 159	
	Storage of clinical samples	130	81.8%
	Devices/tubes/systems used to collect different sample types	127	79.9%
	Timing of collection (for example, in relation to antimicrobial treatment)	126	79.2%

	Sampling site	123	77.4%
	Sampling techniques for different sample types	120	75.5%
	Packaging of clinical samples	119	74.8%
	Other	9	5.7%
**Q9**	**Type of specimens collected (MA)**	n= 216	
	Clinical	198	90.7%
	Foodstuff and animal feed	50	23.1%
	Environmental	40	18.5%
**Q11**	**Species of clinical samples (MA)**	n= 190	
	Farm animals (e.g. cattle, small ruminants, pigs, farmed rabbits, mink and/or poultry)	158	83.2%
	Small animals (dogs and/or cats)	147	77.4%
	Equine	106	55.8%
	Exotic pets (e.g. pet birds, rabbits, rodents, reptiles and/or ornamental fish)	101	53.2%
	Wildlife/Zoo animals	83	43.7%
	Fish	51	26.8%
	Laboratory animals	43	22.6%
**Q12**	**IQA participation (SA)**	n= 190	
	Yes	134	70.5%
	No	56	29.5%
**Q13**	**Type of IQA (MA)**	n= 132	
	Standard Operating Procedures	120	90.1%
	Equipment maintenance and calibration	113	85.6%
	Use of Quality Control strains	113	85.6%
	Good laboratory practice manual	86	65.2%
	Development programs (or competency assessment) for staff	73	55.3%
	Other	17	12.9%
**Q14**	**EQA participation (SA)**	n= 188	
	Yes	112	59.6%
	No	76	40.4%
**Q15**	**Type of EQA (MA)**	n= 111	
	Taking part in national proficiency testing	85	76.6%

	Accreditation from a recognised QA system	76	68.5%
	External audits	75	67.6%
	Taking part in a European proficiency testing scheme	49	44.1%
	Other	8	7.2%
**Q16**	**Composition of laboratory team (MA)**	n= 185	
	Technical staff	157	84.9%
	Veterinary microbiologist(s)	143	77.3%
	Microbiologist(s) (non-veterinary background)	71	38.4%
	Veterinarian/clinician	50	27.0%
	Veterinary pathologist(s)	47	25.4%
	Veterinary clinical pathologist(s)	40	21.6%
	Other (please specify)	13	7.0%
	Veterinary internal medicine specialist(s)	12	6.5%
	Veterinary nurse	9	4.9%
**Q17**	**Professional background of leader (SA)**	n= 185	
	Veterinary microbiologist	103	55.7%
	Veterinarian/clinician	24	13.0%
	Microbiologist (non-veterinary background)	17	9.2%
	Veterinary clinical pathologist	16	8.6%
	Other	13	7.0%
	Veterinary pathologist	7	3.8%
	Veterinary internal medicine specialist	3	1.6%
	Technical staff	2	1.1%
	Veterinary nurse	0	0.0%
**Q18_1**	**Time for bacterial identification (SA)**	n= 185	
	1-2 days	144	77.8%
	3-5 days	37	20.0%
	6-8 days	3	1.6%
	Other	1	0.5%
**Q18_2**	**Time for AST (SA)**	n= 185	
	1-2 days	116	62.7%
	3-5 days	60	32.4%
	6-8 days	8	4.3%
	Other	1	0.5%

Table S 3. *Responses regarding the methodology employed by the participating laboratories for bacterial culture and antimicrobial susceptibility testing. Percentages are based on the*

number of responses to individual questions. MA, multiple answer question; SA, single answer question.

		Number	Percentage
**Q19**	**Service provided (MA)**	n= 178	
	Aerobic culture	174	97.8%
	Anaerobic culture	159	89.3%
	Microaerophilic culture, e.g. *Campylobacter* spp.	137	77.0%
	Culture for organisms growing in 5-10% CO2, e.g. *Haemophilus* spp.	127	71.3%
	Mycoplasma culture	74	41.6%
	Selective culture of target organisms (please specify below)	74	41.6%
	Mycobacterial culture	60	33.7%
	Other (please specify)	43	24.2%

Table S 4. *Responses regarding the methodology employed by the participating laboratories for bacterial culture and antimicrobial susceptibility testing. Percentages are based on the number of responses to individual questions. MA, multiple answer question; SA, single answer question.*

**Q20**	**Identification to species level (SA)**	n= 178	
	Yes	102	57.3%
	In most cases (>50% of cases)	51	28.7%
	In some cases (<50%)	22	12.4%
	No	3	1.7%
**Q21**	**Methods for bacterial identification (MA)**	n= 178	
	Individual biochemical tests (e.g. catalase, oxidase)	137	77.0%
	API kits (bioMérieux or similar)	100	56.2%
	PCR (16s rRNA sequencing or other gene target)	83	46.6%
	MALDI-TOF MS	77	43.3%
	Vitek-1 or Vitek-2	45	25.3%
	Other	20	11.2%
	Trek SENSITITRE	4	2.2%
	BD Phoenix	3	1.7%
	MicroScan Walkaway	1	0.6%
**Q22**	**AST method provided (MA)**	n= 178	

	Disk diffusion (Kirby-Bauer) by default	78	43.8%
	Minimum Inhibitory concentrations (MIC), by default	58	32.6%
	Disk diffusion by request	51	28.7%
	Disk diffusion (Kirby-Bauer) by default and MIC by request	42	23.6%
	MIC by request	27	15.2%
**Q23**	**MIC method provided (MA)**	n= 101	
	Vitek-1 or Vitek-2	40	39.6%
	In house broth microdilution	31	30.7%
	Trek SENSITITRE	28	27.7%
	Gradient test strip (e.g. E-test)	27	26.7%
	In house agar dilution	17	16.8%
	Micronaut AST system	14	13.9%
	Other	7	6.9%
	BD Phoenix	4	4.0%
	MicroScan Walkaway	1	1.0%
**Q24_1**	**Main clinical breakpoints (CBP) used for disc diffusion (SA)**	n= 148	
	Combination of EUCAST/CLSI	61	41.2%
	EUCAST	31	20.9%
	CLSI	26	17.6%
	National guidelines	25	16.9%
	Other	5	3.4%
**Q24_2**	**Main clinical breakpoints used for MIC (SA)**	n= 105	
	Combination of EUCAST/CLSI	50	47.6%
	CLSI	30	28.6%
	EUCAST	16	15.2%
	National guidelines	7	6.7%
	Other	2	1.9%
**Q25**	**Approach when no species-specific CBPs (MA)**	n= 165	
	Use a CBP developed for infection in humans	88	53.3%
	Use a CBP from another organ/organism/animal species	85	51.5%
	Testing but not interpreting antimicrobial susceptibility	51	30.9%

	Report that the isolated organism cannot be tested due to lacking CBP	37	22.4%
	Use epidemiological cutoff (ECOFF) instead of CBP	35	21.2%

Table S 5. *Responses regarding the approaches taken for detecting and interpreting AMR phenotypes. For each phenotype listed, multiple answers were possible.*

**Q26 – What methods do you use for detection and interpretation of AMR phenotypes?**	Disk detection	MIC testing	Chromogenic media	Molecular detection (e.g., PCR or PBP2 agglutination test for MRSA/MRSP)	Send isolates to reference labs	Other
MRSA/MRSP (n= 146)	61.6%	35.6%	28.8%	41.1%	17.8%	3.4%
ESBL-producing *Enterobacterales* (n= 147)	70.1%	40.1%	15.6%	24.5%	12.9%	4.8%
Vancomycin resistant *Enterococcus faecium*/*faecalis* (n= 91)	58.2%	37.4%	12.1%	28.6%	12.1%	5.5%
Inducible clindamycin resistance in Gram-positive bacteria (n=105)	62.9%	36.2%	2.9%	7.6%	6.7%	3.8%
Acquired AmpC β-lactamase-producing *Enterobacterales* (pAmpC) (n=123)	63.4%	42.3%	13.8%	26.0%	12.2%	2.4%
Polymyxin resistance in Gram-negative bacilli (n= 106)	50.9%	43.4%	7.5%	20.8%	8.5%	6.6%
Carbapenemase-producing *Enterobacterales* (CPE) (n= 108)	54.6%	46.3%	23.1%	26.9%	19.4%	7.4%

Table S 6. *Responses regarding reasons for phenotypical screening of various resistant bacteria. For each resistant bacterium listed, multiple answers were possible. Valid answers were considered if a participant interacted with at least one of the questions in a row of the table.*

**Q27 – Do you screen phenotypically for the listed resistant bacteria for any of the reasons shown?**	Do you screen for	Informing antibiotic selection/ therapeutic guidance	Epidemiological surveillance	Infection control	Other
ESBL production(*n*= 165)	46.7%	41.2%	35.2%	23%	4.2%
Acquired AmpC β-lactamase-producing *Enterobacterales* (pAmpC) (*n*= 165)	36.4%	30.9%	28.5%	15.2%	4.2%
Carbapenemase-producing *Enterobacterales* (CPE) (*n*= 165)	32.1%	18.8%	26.7%	13.3%	3.6%
Methicillin-resistant staphylococci (MRS) (*n*= 165)	48.5%	44.8%	36.4%	26.1%	4.2%
Vancomycin-resistant *Enterococcus faecium*/*faecalis* (*n*= 165)	20%	13.9%	17%	8.5%	4.8%
Inducible clindamycin resistance in Gram-positive bacteria (i.e., D-test) (*n*= 165)	26.7%	23.6%	12.1%	10.9%	3.6%

Table S 7. *Responses regarding information used for assessing clinical significance of bacterial isolates obtained from clinical specimens. For each option listed, multiple answers were possible.*

**Q29 - Information used for assessing clinical significance of bacterial isolates obtained from clinical specimens** (n= 157)	Always	Often (60-99% of cases)	Sometimes (30-60% of cases)	Rarely (1-30% of cases)	Never
Knowledge of sampling method/sampling site (*n*= 157)	56.7%	25.5%	7.6%	4.5%	5.7%
Duration/mode of sample transport (*n*= 157)	39.5%	27.4%	13.4%	10.2%	9.6%
Clinical history provided on the submission form (*n*= 157)	51.0%	28.7%	10.2%	4.5%	5.7%
Specimen Gram-stained smear findings (*n*= 157)	24.8%	19.1%	14.6%	25.5%	15.9%
Evidence of inflammation from cytology report (from clinical pathology) (*n*= 157)	15.03%	10.8%	16.6%	24.8%	32.5%

Knowledge of organisms likely to be etiologic agents at the infection site (*n*= 157)	59.2%	21.7%	9.6%	3.8%	5.7%
The amount of growth obtained (*n*= 157)	45.9%	29.3%	13.4%	3.8%	7.6%
The number and relative proportion of distinct organisms cultured (*n*= 157)	45.2%	29.3%	12.1%	5.1%	8.3%
Identification of the organisms at species level (*n*= 157)	47.8%	28.7%	14.0%	2.5%	7.0%
Information obtained from discussion with the clinician in charge of the case (*n*= 157)	22.3%	25.5%	28.0%	15.9%	8.3%
Other (*n*= 157)	2.5%	3.2%	1.9%	4.5%	11.5%

Table S 8. *Responses on number of bacterial isolates typically selected for antimicrobial susceptibility testing from normally sterile and non-sterile body sites.*

**Q30 - Number of isolates typically selected for antimicrobial susceptibility testing from one sample**	All bacterial isolates representing all colony types	Pure growth isolates only	Up to max 3 isolates	Up to max 2 isolates	Other
Normally sterile body sites (e.g., blood, CSF, synovial fluid) (*n*= 154)	31.2%	31.8%	12.3%	15.6%	9.7%
Non-sterile body sites (e.g., skin, mucosal surfaces) (*n*= 154)	4.5%	23.4%	36.4%	25.3%	10.4%

Table S 9. *Laboratory responses regarding inclusion in the results report of comments concerning the clinical significance of the isolated organisms.*

**Q28 - Comments regarding the clinical significance of the isolated organisms by providing an estimate on their likely role**	Yes	No	On “ad hoc” basis	Other
Likely commensal/resident flora (n= 157)	41.4%	28.7%	25.5%	4.5%
Likely opportunistic pathogenic (n= 157)	30.6%	36.3%	29.3%	3.8%
Clinically significant (n= 157)	48.4%	29.9%	17.8%	3.8%

Table S 10. *Responses regarding inclusion in the results report of recommendations/comments.*

**Q31 - Do you as a default include the following when sending your culture and antimicrobial susceptibility results to the requesting veterinarian?**	Always	Often (60-99%)	Sometimes (30-60%)	Rarely (1-30%)	Never

Indicate what antimicrobials are suitable for different infection types and/or body sites (*n*= 157)	25.5%	18.5%	7.0%	10.8%	38.2%
Indicate suitable antimicrobials for organisms for which AST is difficult to perform (e.g., anaerobic organisms) (*n*= 157)	21.0%	17.8%	12.1%	17.2%	31.8%
Indicate that for MRSA/MRSP/ESBL carriers, antibiotic treatment is not indicated (*n*= 156)	23.1%	12.8%	12.2%	12.2%	39.7%
Show breakpoints that were applied (e.g., CLSI-VET08, 2018) (*n*= 156)	22.4%	7.7%	8.3%	10.3%	51.3%
Specify when results are "Not Interpretable" due to the lack of an agreed breakpoint for certain organisms/antimicrobials combination (*n*= 157)	29.9%	8.3%	10.8%	15.3%	35.7%

Table S 11*. Laboratory responses on the approach taken by laboratories to encourage prudent antimicrobial use when reporting antimicrobial susceptibility testing results. Multiple answers were possible from each respondent.*

**Q32 - When reporting antimicrobial susceptibility testing results, what approach do you generally take to encourage prudent antimicrobial use?**	n= 157	%
Selective reporting [I.e., suppress Highest Priority Critically Important Antimicrobials (HPCIA)]	62	39.5%
Cascade reporting (i.e., report AST results for only one drug within a certain class)	25	15.2%
Group antimicrobials by "tier" OR 1st line, 2nd line, etc.; with a comment indicating that higher	18	11.5%
Indicate which antimicrobials are suitable according to the site of infection and antibiotic pen	33	21.0%
Indicate when topical and not systemic usage would be appropriate	21	13.4%
Indicate "Doubtful clinical significance, no treatment is indicated"	33	21.0%
Indicate "Discussion with clinician required?"	26	16.6%
No specific approach taken	59	37.6%
Other (please specify)	18	11.5%

Table S 12. *Responses regarding Minimum Inhibitory Concentration (MIC) specific information (e.g., clinical interpretation only, with or without clinical breakpoints used for interpretation) provided on the laboratory results reports.*

**Q33 - MIC specific information provided on the results report**	Always	Upon request	N/A*	
S, R, I only (without MIC values) (n= 96)	47.9%	11.5%	40.6%	n= 96
S, R, I and the MIC value without breakpoints (n= 96)	36.5%	30.2%	33.3%	n= 96
S, R, I and the MIC value with the breakpoint values (n= 95)	25.3%	29.5%	45.3%	n= 95
Other (n= 24)	25.0%	12.5%	62.5%	n= 24

* Not applicable (typically because repondent did not use MIC testing for AST)

Table S 13. *Responses on availability of data management systems.*

**Q35 – Please indicate what data management system do you use in your laboratory**	Yes	No	N/A
Do you use a computerised system for sample recording/accessioning? (n= 151)	86.1%	13.9%	1.3%
Do you use a computerised system for reporting? (n= 151)	86.0%	14.0%	2.0%
Are you able to store and extract culture and susceptibility testing results? (n= 151)	91.3%	8.7%	2.0%
Can you extract data to analyse antimicrobial resistance trends? (n= 151)	77.0%	23.0%	3.4%

Table S 14. *Responses on participation in pathogen surveillance schemes.*

**Q36 - Do you take part in any pathogen surveillance schemes? (SA)**	Yes	No
Antimicrobial resistance, for farm species (n= 153)	53.6%	46.4%
Antimicrobial resistance, for companion animals (n= 152)	40.1%	59.9%
Salmonella reporting and typing (n= 152)	59.2%	40.8%
Zoonotic pathogens surveillance (n= 153)	44.4%	55.6%
Other (n= 60)	18.3%	81.7%

Table S 15. *Laboratory responses on the need for guidelines development for veterinary clinical microbiology laboratories.*

**Q37 - If new specific guidelines for veterinary clinical microbiology laboratories are developed, please score which you consider the most important aspects that will make a difference in the quality of the results you provide?**	**1-not important**	**2**	**3**	**4**	**5-highly important**
Provide common guidelines for bacterial culture, isolation and identification (*n*=153)	2.6%	5.2%	15.0%	23.5%	53.6%
Issue guidelines for interpreting and reporting clinical significance of bacterial cultures (*n*=153)	2.0%	5.2%	13.7%	28.1%	51.0%
Recommend "preferred" guidelines for interpreting and reporting antimicrobial susceptibility results (CLSI-VAST or EUCAST) (*n*=153)	3.3%	2.6%	10.5%	23.5%	60.1%
Issue guidelines for what to do in the absence of breakpoints (*n*=153)	3.9%	3.3%	12.4%	22.9%	57.5%
Issue guidelines for surveillance of antimicrobial resistance in veterinary isolates (*n*=153)	2.0%	8.5%	13.1%	26.1%	50.3%
Provide expert rules on intrinsic resistance and exceptional phenotypes for veterinary pathogens (*n*=153)	3.03%	5.2%	20.9%	22.2%	48.4%
Provide guideline for the detection of resistance mechanisms including specific resistances of clinical and/or epidemiological importance (*n*=153)	2.0%	9.8%	13.7%	27.5%	47.1%
